# Unraveling Unique Effect of Ergosterol on Lipid Membranes, and What We Can Do with the Future STS

**DOI:** 10.1063/4.0000937

**Published:** 2025-10-27

**Authors:** Shuo Qian, Gergely Nagy, Piotr Zolnierczuk, Eugene Mamontov, Robert Standaert

**Affiliations:** Oak Ridge National Laboratory, Oak Ridge, TN, USA

## Abstract

Ergosterol, a critical component of fungal membranes, has traditionally been thought to influence membrane properties in much the same way as cholesterol because of their similar structures. However, our neutron scattering study challenges that view, revealing key differences in how these sterols interact with and impact lipid bilayers.

Our neutron diffraction experiments show that ergosterol settles much closer to the membrane surface than cholesterol, resulting in only slight changes to membrane thickness—quite the opposite of cholesterol’s pronounced condensing effect. Additionally, our neutron spin echo measurements indicate that ergosterol can either stiffen or soften the membrane depending on its concentration, a versatility not seen with cholesterol, which consistently makes membranes more rigid. Even more intriguing, quasi-elastic neutron scattering data uncovers that ergosterol significantly alters lipid motion; rather than the continuous diffusion observed in pure lipid or cholesterol-containing membranes, ergosterol introduces a stepwise jump diffusion in lipid.

Together, these insights reveal ergosterol as a unique membrane component with effects that differ markedly from those of its animal counterpart.

